# Sciatic lateral popliteal block with clonidine alone or clonidine plus 0.2% ropivacaine: effect on the intra-and postoperative analgesia for lower extremity surgery in children: a randomized prospective controlled study

**DOI:** 10.1186/1471-2253-12-2

**Published:** 2012-02-01

**Authors:** Kalliopi Petroheilou, Stavros Livanios, Nikolaos Zavras, John Hager, Argyro Fassoulaki

**Affiliations:** 1 General Children's Hospital, Department of Anesthesiology, 8. Hippocratous Str, P Penteli, 15236, Greece; 2 General Children's Hospital, Department of Surgery, 8 Hippocratous Str, P Penteli, 15236, Greece; 3 General Children's Hospital, Department of Orthopaedics, 8 Hippocratous, Str, P Penteli, 15236, Greece; 4 Aretaieio Hospital, University of Athens, Medical School, Department of Anesthesiology, 76 Vassilissis Sofias Ave, Athens 11528 Greece

## Abstract

**Background:**

The effect of adding clonidine to local anesthetics for nerve or plexus blocks remains unclear. Most of the studies in adults have demonstrated the positive effects of clonidine on intra- and postoperative analgesia when used as an adjunctive agent or in some cases as a single to regional techniques. In the pediatric population, there are only few trials involving clonidine as an adjunct to regional anesthesia, and the analgesic benefits are not definite in this group of patients. The evidence concerning perineural administration of clonidine is so far inconclusive in children, as different types and volume of local anesthetic agents have been used in these studies. Moreover, the efficacy of regional anesthesia is largely affected by the operator's technique, accuracy and severity of operation.

**Methods:**

The use of clonidine alone or combined with 0.2% ropivacaine for effective analgesia after mild to moderate painful foot surgery was assessed in 66 children, after combined sciatic lateral popliteal block (SLPB) plus femoral block. The patients were randomly assigned into three groups to receive placebo, clonidine, and clonidine plus ropivacaine. Time to first analgesic request in the groups was analyzed by using Kaplan-Meier and the log-rank test (mean time, median time, 95% CI).

**Results:**

In our study, clonidine administered alone in the SLPB seems promising, maintaining intraoperatively the hemodynamic parameters SAP, DAP, HR to the lower normal values so that no patient needed nalbuphine under 0.6 MAC sevoflurane anesthesia, and postoperatively without analgesic request for a median time of 6 hours. In addition, clonidine administered as adjuvant enhances ropivacaine's analgesic effect for the first postoperative day in the majority of children (p = 0.001). Clonidine and clonidine plus ropivacaine groups also didn’t demonstrate PONV, motor blockade, and moreover, the parents of children expressed their satisfaction with the excellent perioperative management of their children, with satisfaction score 9.74 ± 0.45 and 9.73 ± 0.70 respectively. On the contrary all the patients in the control group required rescue nalbuphine in the recovery room, and postoperatively, along with high incidence of PONV, and the parents of children reported a low satisfaction score (7.50 ± 0.70).

**Conclusions:**

Clonidine appears promising more as an adjuvant in 0.2% ropivacaine and less than alone in the SLPB plus femoral block in children undergoing mild to moderate painful foot surgery, with no side effects.

**Trial registration:**

ClinicalTrials.gov, ISRCTN90832436, (ref: CCT-NAPN-20886).

## Background

The clinical importance of clonidine in peripheral blocks of children and adults for effective intra - and postoperative analgesia is controversial and subjected to an ongoing debate.

In adults, the majority of the studies (reviews and meta-analyses randomized trials) have demonstrated the positive effects of clonidine on intra - and postoperative pain relief, when used as an adjunctive agent to peripheral nerve blocks [1-5].

Moreover, clonidine alone may have a potential role to produce analgesia when used for regional techniques in adults [6-8].

In the pediatric population, there are only few trials involving clonidine as an adjunct to peripheral blocks, and the analgesic benefits are not definite in this group of patients. The clinical benefits of clonidine in moderate and severe painful surgeries remain questionable [9,10] or potentially with limited analgesia [11,12]. However, in a pediatric pilot study, a trend has been demonstrated for better postoperative analgesia following peripheral administration of clonidine compared with central use [13]. Moreover, there are controversies if caudal or systemic administration of clonidine can also enhance regional block in children [14-16].

Our hypothesis was that clonidine alone or combined with 0.2% ropivacaine could produce a long lasting block after foot and ankle surgery, adequate for the first postoperative day.

The aim of the present randomized, prospective, controlled study was to determine whether the use of clonidine alone or combined with 0.2% ropivacaine, as single shot, in the sciatic lateral popliteal block (SLPB) plus femoral block, could provide adequate intra-and postoperative analgesia in children undergoing mild to moderate painful foot and ankle surgery.

## Methods

### Patients, Randomization and Blindness

Between January 2009 to May 2010, children, ASA physical status I and II, aged 5-14 years were scheduled for elective mild to moderate painful foot and ankle surgery. According to the power analysis, 66 children should be studied. During the study, eleven children with neurologic or neuromuscular disease, problems in communication, skin infection at the site of needle insertion, or children's parents refusal were excluded. We used a computer generated table program to produce random numbers. We made a choice of twenty one (n = 21) random numbers from 1 till 66 in order to be in the control group, twenty three (n = 23) random numbers remained from the first choice to be in the clonidine group, and the rest twenty two (n = 22) numbers to be in the clonidine plus 0.2% ropivacaine group. The investigators were blind to the group assignment. The placebo or the treatment solutions were prepared by the pharmacy and supplied to the Department of Anesthesia in syringes labeled with predetermined code for each solution. There were two syringes for the SLPB and the femoral block respectively. In the SLPB, the syringes contained a) for the control group isotonic saline 10 ml plus 0.25 ml/kg and saline 0.13 ml/kg, b) for the clonidine group isotonic saline 10 ml plus 0.25 ml/kg, and clonidine 2 μg/kg (0.13 ml/kg) respectively. c) finally in the clonidine plus 0.2% ropivacaine group the syringes contained 0.2% ropivacaine 10 ml plus 0.25 ml/kg (maximum 25 ml) and clonidine 2 μg/kg (0.13 ml/kg) respectively. Similarly in the femoral block the syringes contained a) for the control group isotonic saline: 0.4 ml/kg and 0.065 ml/kg respectively, b) for the clonidine group isotonic saline 0.4 ml/kg and clonidine 1 μg/kg (0.065 ml/kg) respectively, and c) for clonidine plus 0.2% ropivacaine group 0.2% ropivacaine 0.4 ml/kg and clonidine μg/kg (0.065 ml/kg) respectively. The maximum dose of 0.2% ropivacaine was decided to be 3.5 mg/kg and for clonidine 3 μg/kg.

The children were enrolled in the study after approval of The Scientific Ethics Committee (General Children's Hospital, Penteli, Athens). Preoperatively, we explained to parents our intervention treatment. After understanding and accepting the alternative treatments by randomization, without coercion or manipulation, written informed consent was obtained in the card anesthesia.

### Anesthetic technique

No premedication was used. General anesthesia was induced with sevoflurane 8% and maintained with sevoflurane adjusted up to 2.6% (1.3 MAC) to control the hemodynamic responses in an oxygen air mixture in a 1/2 ratio and followed by a combined SLPB plus femoral block. Regional blocks were performed by the two anesthesiologists and operations by two surgeons. After the performance of nerve blocks, all patients received cis-atracurium (0.1 mg/kg) and trachea was intubated. Nalbuphine infusion 0.03 mg/kg/h was administered in all patients over 15 min period after the performance of nerve blocks. Additional nalbuphine (0.03-0.09 mg/kg/h) was administered during surgery in the case of persistent tachycardia and hypertension (defined as values 15% more than baseline) in patients that did not respond to increased inspired sevoflurane concentration. Heart rate (HR), arterial systolic and diastolic pressure (SAP, DAP), S_P_O_2_, were recorded preoperatively (baseline, time 0) and intraoperatively, plus PetCO_2_, every 15 min until the end of the operation. The total amount of nalbuphine was also recorded at the end of the operation.

### Regional Anesthesia

In the anesthetized children in the supine position, the SLPB [17-19] was performed, using 100 mm or 50 mm, 21 gauge insulated stimulated needle (Stimuplex B, Braun Medical, Bethlehem, PA). The sciatic nerve was localized by means of a nerve stimulator, with a stimulating current ranged between 0.45-0.55 (< 0.6)mA, eliciting strong inversion (deep peronial DP, branch of the CPN plus TN) or plantar flexion (tibial nerve TN) or dorsiflexion (common peronial nerve CPN) of the foot. An additional femoral block [18] became necessary for the use of tourniquet in the area around the thigh. After the performance of blocks a pneumatic tourniquet at 150 mmHg was applied to the mid-thigh.

### Postoperative analgesic protocol

Postoperative analgesia was assessed by by means of a color analogue scale (CAS) [20]. Patients with mild or moderate postoperative pain (CAS score > 30 to 45 mm and 46 to 55 mm respectively) received nalbuphine 0.2 mg/kg and 0.3 mg/kg respectively.

### Data

In the recovery room, and the orthopedic ward the following data were collected by the anesthesiologists: (a) time to first analgesic request of nalbuphine after the surgery; (b) pain CAS score at rest, was assessed in the recovery room (time:0), 2, 4, 6, 8, 18, 24 hours postoperatively, and the tourniquet pain (time:0). CAS ranging from 0 to 100 mm, with 100 mm being the worst pain imaginable; (c) total number of rescue nalbuphine doses and the total amount of nalbuphine for the 24 hours observational period; (d) duration of sensory block by testing sensation (pin prick) in the distribution area of the sciatic nerve postoperatively. The degree of sensory block was classified as follows: 0: normal sensation, 1: blunted sensation (analgesia), 2: absence of sensation (anesthesia); (e) duration of motor block postoperatively. Motor block was assessed with voluntary plantar flex (TN), or dorsiflex (CPN) patient's foot and was classified as follows: 0: normal movement, 1: decreased movement (incomplete motor block), and 2: no movement (complete motor block); (f) restless as dichotomous (yes or no); (g) incidence of nausea and/or vomiting; (h) sedation level using a four - point scale (0: awake, 1: drowsy, 2: asleep but easily arousable with verbal command, 3: asleep but not easily arousable, only by tactile stimulation), was checked; (i) side effects from the SLPB; (k) satisfaction score was assessed by the parents of children the second postoperative day , using a numerical scale ranged from 0 to 10.

### Statistical analysis

Ordinal data were presented as mean ± SD or ± SE as indicated. The one way ANOVA and the test Bonferroni were used to compare the absolute values of variables between the groups and pair wise respectively. The comparison of categorical parameters was analyzed by using Chi-Square test or Fisher's exact test. Time to rescue analgesic administration in the groups was analyzed by using Kaplan-Meier and the log-rank test (mean time, median time, 95% CI). A power analysis was performed by statistic program G*Power vr 3-1-2, using the mean time period from the end of anesthesia to the first analgesic request. A one way ANOVA analysis, sample sizes of 21, 23, and 22 were obtained from three groups whose means were to be compared. The total sample of 66 subjects achieved a 100% power to detect differences among the means versus the alternative of equal means using an F test with a 0.05 significance level. The common standard deviation within a group is assumed to be 8,00. All tests are two-sided. A p < 0.05 value was considered to be statistically significant. The statistical analysis was performed with the SPSS, version13.00 (SPSS Inc, Chicago, IL).

## Results

**Figure 1:**
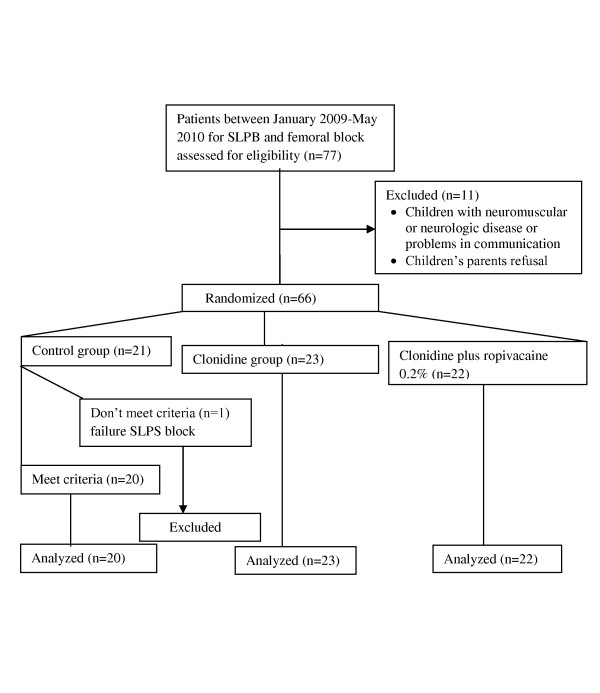
**Flow diagram of the study**.

Of the 66 patients studied in the analysis, one more patient in the control group was also excluded because of failure to localize the sciatic nerve with stimulator guidance (Figure 1). The groups were comparable with respect to demographic data (age in years, weight, and sex), the type of operation, and the total anesthesia time (Table 1). A remarkable similarity of attempts to the ease of sciatic nerve localization was observed (p = 0.503).

**Table 1:** Demographic and surgical data of the study population

	CTL (n = 20)	**CLON**. (n = 23)	CLON. ROP (n = 22)	p- value
Age (years).	9 (± 2)	10 (± 2)	10 (± 2)	0.254

Sex (M|F)	7/13	13|10	10|12	0.466

Weight (kg)	32.6 (± 10.4)	35.9 (± 9)	35 (± 11.6)	0.618

Time of anaesthesia (min)	122.25 (± 23.97)	120.22 (± 24.9)	127.5 (± 23)	0.25

Mild Clubfoot	12	13	12	0.5

Achilles' lengthening	8	10	10	0.126

Data are presented as mean ± SD

Intraoperatively in clonidine, and clonidine plus ropivacaine groups the hemodynamic parameters SAP, DAP, HR were maintained to the lower normal values so that no patient needed nalbuphine, under 0.6 MAC sevoflurane anesthesia. On the contrary, in the control group, increased sevoflurane concentration (MAC up to1.3), and nalbuphine infusion (0.09 mg/kg/h, mean value: 5.097 ± 3.33), were required in the patients, to maintain the parameters to the lower normal values until the end of the operation.

**Figure 2:**
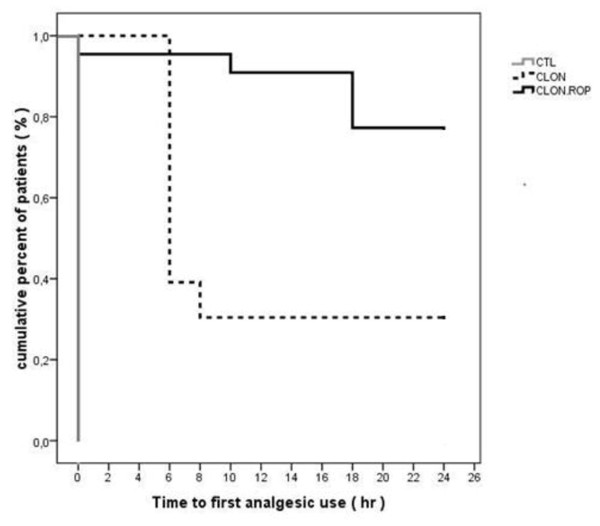
**Kaplan-Meier analysis**. Estimation of survival analysis of the time from the end of anesthesia to the first analgesic request in the Control, Clonidine and Clonidine plus 0.2% Ropivacaine groups.

Postoperatively, the Kaplan- Meier analysis (Figure 2), shows the time from the end of anesthesia to the first analgesic request which differed significantly between the three groups (overall p < 0.0005). Particularly, the patients in the clonidine plus ropivacaine group had a significantly longer time to first analgesic request compared to clonidine group (Figure 2), [mean time: 21.5 hours, SEM: 1.26, SD:5.90, 95% CI:19.0-23.9 versus 11.6 hours, SEM:1.74 SD: 8.33, 95% CI: 8.2-15.1, median time: 24 hours, range:0-24, 95% CI: 21.6-26.4 versus 6 hours, range:6-24, 95% CI: 5.6-6.4], (p = 0.001). On the contrary to the control group that all patients required rescue nalbuphine in the recovery room (time:0), the patients in the clonidine group had a longer mean time to first rescue nalbuphine (p < 0.0005) (Figure 2).

**Figure 3:**
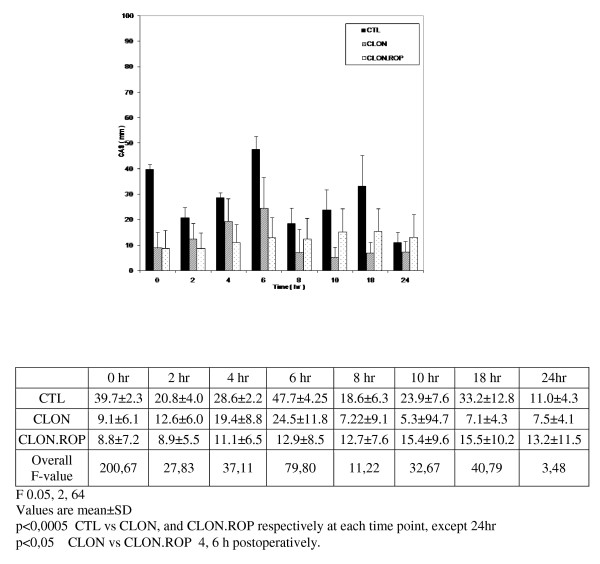
**CAS pain between groups**. The mean pain CAS score (mm) at rest in the Control, Clonidine, and Clonidine plus 0.2% Ropivacaine groups at 0, 2, 4, 6, 8, 10, 18, 24 h time points after surgery.

The mean pain CAS score also differed significantly between the three groups (overall p < 0.0005), (Figure 3). In the recovery room (time 0) the mean pain CAS score was similar in normal values concerning the clonidine and clonidine plus ropivacaine groups and significantly lower than in the control group (p < 0.0005) (Figure 3). No patient in the clonidine and clonidine plus ropivacaine groups complained for tourniquet pain, indicating the success of the femoral block as a supplement of perioperative analgesia. On the contrary, all patients in the control group complained for tourniquet pain.

In the ward the mean pain CAS score in clonidine and clonidine plus ropivacaine groups was lower than in the control group (p < 0.0005), while at 4 and 6 hours postoperatively the mean pain CAS score in clonidine plus ropivacaine group was lower than in the clonidine group (p < 0.05), (Figure 3).

**Figure 4:**
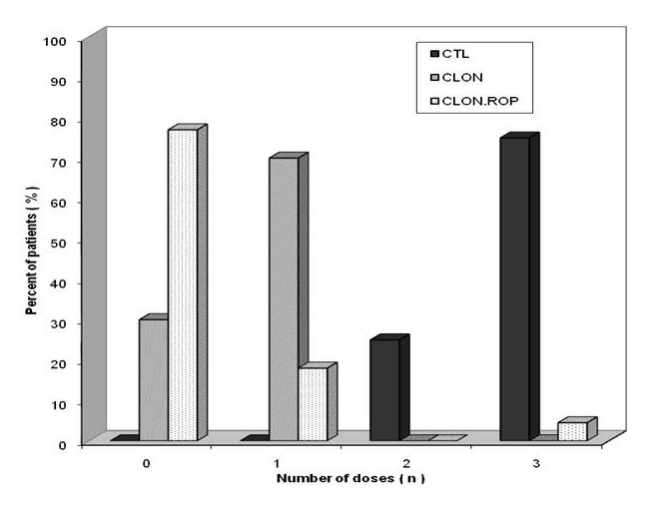
**Rescue doses of Nalbuphine**. Rescue doses of Nalbuphine in the Control, Clonidine and Clonidine plus 0.2% Ropivacaine groups at 0 up to 24 h after surgery.

According to the protocol, a rescue dose of 0.2 mg/kg nalbuphine was given as follows: In the clonidine plus ropivacaine group four patients required one rescue dose and one required 3 doses. In the clonidine group, 16 patients required one rescue dose (Figure 4). In the control group 15 patients required 3 rescue doses of nalbuphine and 5 required 2 doses. However, nine of them required a rescue dose of 0.3 mg/kg, because of a pain CAS score more than 45 mm at 6 hours postoperatively.

The sensory block in the distribution of all the area of the sciatic nerve or/the CPN, and in the area of the femoral nerve, in the clonidine plus ropivacaine group lasted 16.45 ± 5.81 hours, and 8.6 ± 2.6 hours respectively. No patient in the clonidine group presented sensory block.

Furthermore, most children of clonidine plus ropivacaine, and clonidine groups, eliciting plantar flexion or strong inversion of the foot presented better postoperative analgesia and required fewer rescue doses of nalbuphine compared to children eliciting dorsiflexion of the foot (Chi-Square = 18.66, p = 0.003).

None of the patients in clonidine, and clonidine plus ropivacaine groups presented motor block.

In the recovery, all the patients of clonidine and clonidine plus ropivacaine groups were calm versus 9 patients of the control group (p < 0.005). In the ward during the 24 hours postoperative observation period, 10 of the 20 patients of control group presented PONV once, but none in the clonidine/clonidine plus ropivacaine groups (p < 0.005).

In clonidine plus ropivacaine group 6 of the 22 patients were sedated (level 1) lasting one hour versus sedation level 0 in all patients of control and clondine groups. In this study, none of the patients presented side effects due to SLPB.

During the second postoperative day, the parents of children, of control, clonidine and clonidine plus ropivacaine groups expressed a satisfaction mean score of 7.50 ± 0.70, 9.74 ± 0.45, 9.73 ± 0.70 respectively (p < 0.0005) about the perioperative management of their children.

## Discussion

Our results demonstrate that intra- and postoperative analgesia using combined SLPB plus femoral block either with clonidine alone or clonidine plus ropivacaine for mild to moderate painful foot and ankle surgery in children is effective and safe.

In our study, clonidine administered alone in the SLPB seems promising, maintaining intraoperatively the hemodynamic parameters SAP, DAP, HR to the lower normal values so that no patient needed nalbuphine under 0.6 MAC sevoflurane anesthesia, and postoperatively without analgesic request for a median time of 6 hours. In addition, clonidine administered as adjuvant enhances ropivacaine's analgesic effect for the first postoperative day in the majority of children (p = 0.001). On the contrary, the patients of the control group required high doses of nalbuphine given as continuous intravenous infusion intraoperatively, and in multiple bolus doses postoperatively. Moreover, assessing the cardiovascular parameters this group required high concentration of sevoflurane resulting in a high incidence of PONV [21,22].

Our results also show that in most children, eliciting plantar flexion or strong inversion of the foot, had better quality of analgesia requiring fewer rescue doses of nalbuphine. This is ought to the better spread of local anesthetic solution, when the injecting needle tip presumably lies between the common peroneal nerve laterally and the tibial nerve medially (plantar flexion of the foot), or very close to the trunk of the sciatic nerve before its division (strong inversion of the foot).

The peripheral analgesic effect of clonidine appears clear in a few adults trials, via a local mechanism (drug interaction, action on peripheral a_2_-receptors, or both) after peripheral blocks or intra-articularly. This is supported by the lower clonidine plasma concentrations in block treatment group compared to the systemic treatment group [2,3,7,23,24]. Particularly, in a study clonidine without local anesthetic given through an interscalene catheter provided better analgesia compared with the systemic administration of the same dose [7]. In children, the evidence concerning perineural administration of clonidine is so far inconclusive, as different types and volume of local anesthetics agents have been used in the pediatric studies [9-13]. Moreover, efficacy of regional anesthesia is largely affected by the operator's technique and accuracy and severity of operation.

Therefore, we decided in this study to investigate clonidine effect as an adjuvant in ropivacaine or alone administered in the SLPB in a definite scheme, assuming that intravenous or intramuscular clonidine may be associated with more side effects (e.g. hypotension, bradycardia) in children. Nevertheless, in this study a systemic effect of clonidine cannot be excluded, indicating the calmness without postoperative delirium, as well as without incidence of shivering and PONV, that could be ought to the use of sevoflurane, in the patients of both SLPB. These effects could make clonidine a useful oral premedication medicine in children [25,26].

In our study the lack of sensory and motor block by clonidine alone may be attributed to the small predetermined doses [6]. Furthermore, it was not found motor block in clonidine plus ropivacaine group, confirming previous studies that demonstrate that 0.2% ropivacaine provides preservation of motor function [27].

The analgesic action of clonidine in the SLPB may be explained by the progressive reduction of the myelin layers from the proximal to the distal nerve fibers as in the sciatic nerve at the popliteal fossa, rendering the clonidine more accessible. On the contrary, the denser myelin layers proximal fibers, like the brachial plexus, render clonidine added to 0.2% ropivacaine less accessible, with confined analgesic action but 0.2% ropivacaine alone has also even less analgesic effect [12]. A direct local anesthetic action of clonidine on neural transmission is the most probable suggested mechanism, mediated by primary afferent terminal **α**_2_- adrenergic nociceptors of subtypes C (the more numerous at the primary afferent terminals than on the axons), and 2A (peripheral and central sites), resulting in a decrease in cAMP in the primary afferents and inhibition of PGE_2 _hyperalgesia [23,28-31].

Consequently, the effect of clonidine, administered alone at the distal nerve blocks or added to ropivacaine, seems promising in the less painful foot surgeries like Achilles lengthening and mild clubfoot of our patients. However, the effect of clonidine in severe painful foot, ankle and knee surgery remains questionable [10] or potentially with confined analgesia [11].

It has been suggested that an effective block by a large dose of long acting local anaesthetic may reduce the incidence of tourniquet pain by blocking C and Aδ fibres [32]. However, in our study the limited duration of the foot surgery and the added clonidine in the femoral block demonstrated the absence of incidence of tourniquet pain [32] in clonidine group and clonidine plus 0.2% ropivacaine group, while all the patients in the control group complained of mild tourniquet pain with CAS score 30 mm- 35 mm in the area of its application. The tourniquet pain was also differentiated by the more intense surgical pain with CAS score > 30-55 mm.

In this study, one could be claimed to the role of 0.2% ropivacaine alone or systemic clonidine. However, adult studies have shown a more clearly peripheral effect of clonidine versus its systemic effect [2,3,7,24] indicating a challenge to us for further investigation of clonidine administered in distal peripheral blocks in children. Our results demonstrated the superior analgesic effect of clonidine administered as adjuvant for 0.2% ropivacaine in SLPB in comparison to previous paediatric studies using 0.2% ropivacaine alone in the popliteal fossa block for similar painful foot procedures [33] or in the axillary brachial plexus block [12].

## Conclusions

In conclusion, clonidine appears promising more as an adjuvant in 0.2% ropivacaine and less than alone in the SLPB plus femoral block with regard to mild, moderate intraoperative and postoperative pain management, along with the absence of incidence of PONV, and the high parental satisfaction, in children undergoing foot surgery. Considering the lack of similar studies in children using this technique in this single shot scheme, further investigation is needed to elucidate the definite role of clonidine, and the utility of SLPB for effective analgesia and patient outcome after foot, ankle and knee surgery.

## Competing interests

The authors declare that they have no competing interests.

## Authors' contributions

KP conceived the study design, collected the data, and wrote the manuscript. SL contributed to study design and manuscript preparation. NZ performed data analysis. JH has been involved in revising the manuscript. AF contributed to final protocol development and revised the manuscript. All authors approved the final manuscript.

## Pre-publication history

The pre-publication history for this paper can be accessed here:

http://www.biomedcentral.com/1471-2253/12/2/prepub
